# Disruption of Hepatic Sinusoidal Homeostasis Leads to Hepatopulmonary Syndrome

**DOI:** 10.1111/jcmm.70585

**Published:** 2025-05-08

**Authors:** Jiaxin Chen, Yangkun Guo, Xiaoxun Zhang, Dengcheng Zhou, Yongfang Zhou, Qiong Pan, Jin Chai, Jinhang Gao

**Affiliations:** ^1^ Department of Gastroenterology, Lab of Gastroenterology and Hepatology West China Hospital, Sichuan University Chengdu China; ^2^ Department of Gastroenterology, Institute of Digestive Disease of PLA, Cholestatic Liver Diseases Center and Center for Metabolic Associated Fatty Liver Disease The First Affiliated Hospital (Southwest Hospital), Third Military Medical University (Army Medical University) Chongqing China; ^3^ Key Laboratory of Birth Defects of MOE, State Key Laboratory of Biotherapy, West China Second Hospital, College of Life Sciences Sichuan University Chengdu China; ^4^ Department of Respiratory Care West China Hospital of Sichuan University Chengdu Sichuan China

**Keywords:** intrapulmonary vascular dilatation, liver cirrhosis, liver diseases, liver dysfunction, portal hypertension

## Abstract

Hepatopulmonary syndrome (HPS) is a pulmonary vascular complication of liver disease and/or portal hypertension. HPS manifests as impaired gas exchange and hypoxemia due to intrapulmonary vascular dilatations and shunts. In response to primary liver disease, the abnormal adaptation of respiratory epithelial cells, pulmonary endothelial cells and immune cells leads to pulmonary microenvironment disequilibrium and HPS. In this review, we explore the pathophysiologic mechanisms of HPS, including vascular dilation, angiogenesis and alveolar dysfunction. The liver is the primary contributor to HPS, and liver transplantation is the only treatment that generally reverses HPS. We then discuss how disruption of hepatic sinusoidal homeostasis may impact the progression of HPS, mainly focusing on hepatocytes, cholangiocytes, LSECs and macrophages. As HPS occurs more commonly in advanced liver cirrhosis, we also discuss that normalisation of liver dysfunction and portal hypertension is crucial for the resolution of HPS. In conclusion, liver‐targeted therapies may be effective in treating HPS.

## 1 Introduction

Hepatopulmonary syndrome (HPS) is a pulmonary vascular complication commonly triggered by liver cirrhosis and portal hypertension. HPS is characterised by intrapulmonary vascular dilatations (IPVDs) and consequent abnormal oxygenation [1]. HPS affects 10%–30% of patients with liver cirrhosis, including children and liver transplantation candidates, and is associated with significantly increased mortality rates and worsened quality of life [2, 3, 4, 5, 6, 7]. Patients with early HPS are often asymptomatic, with typical manifestations emerging as the condition progresses, including digital clubbing, cyanosis, diffuse telangiectasias, platypnea and orthodeoxia [1]. The presence of liver disease and/or portal hypertension, evidence of IPVDs detected by contrast‐enhanced transthoracic echocardiography and elevated alveolar–arterial oxygenation gradient determined by arterial blood gas analysis comprise the triad of diagnostic criteria [1]. Despite extensive research on medical options for HPS, liver transplantation remains the only curative option [1]. Therefore, further research on potential therapeutic targets and novel options to either delay HPS progression or cure the condition is needed.

Over the past 30 years, experimental models, especially common bile duct ligation (CBDL) induced liver cirrhosis in rats and mice, have provided insights into the pathogenesis and potential therapeutic targets for HPS [8, 9]. Studies in the preclinical models revealed that several significant alterations in the pulmonary microenvironment contribute to abnormal gas exchange and hypoxemia in HPS [9, 10]. However, previous research has primarily focused on targeting the lung, yielding undesirable results [9]. For instance, Sorafenib was validated in preclinical settings but has shown limited clinical benefit [11, 12]. Additionally, most human studies on HPS include a small number of patients, resulting in contradictory outcomes or unclear benefits [13, 14]. Although the presence or severity of HPS is not closely associated with the extent of the underlying liver disease, liver transplantation is the only curative treatment, providing a rationale for liver‐targeted therapies in HPS [15]. Given that the liver is the fundamental contributor, we propose a new perspective for HPS treatment by targeting the disruption of hepatic sinusoidal homeostasis, liver dysfunction and portal hypertension. This review provides an overview of the underlying pathophysiologic processes responsible for alterations and disequilibrium of the pulmonary microenvironment in HPS. Unlike other reviews, we focus on providing an understanding of mechanisms and potential targets related to the liver in HPS.

## 2 Pulmonary Microenvironment Disequilibrium

**FIGURE 1:**
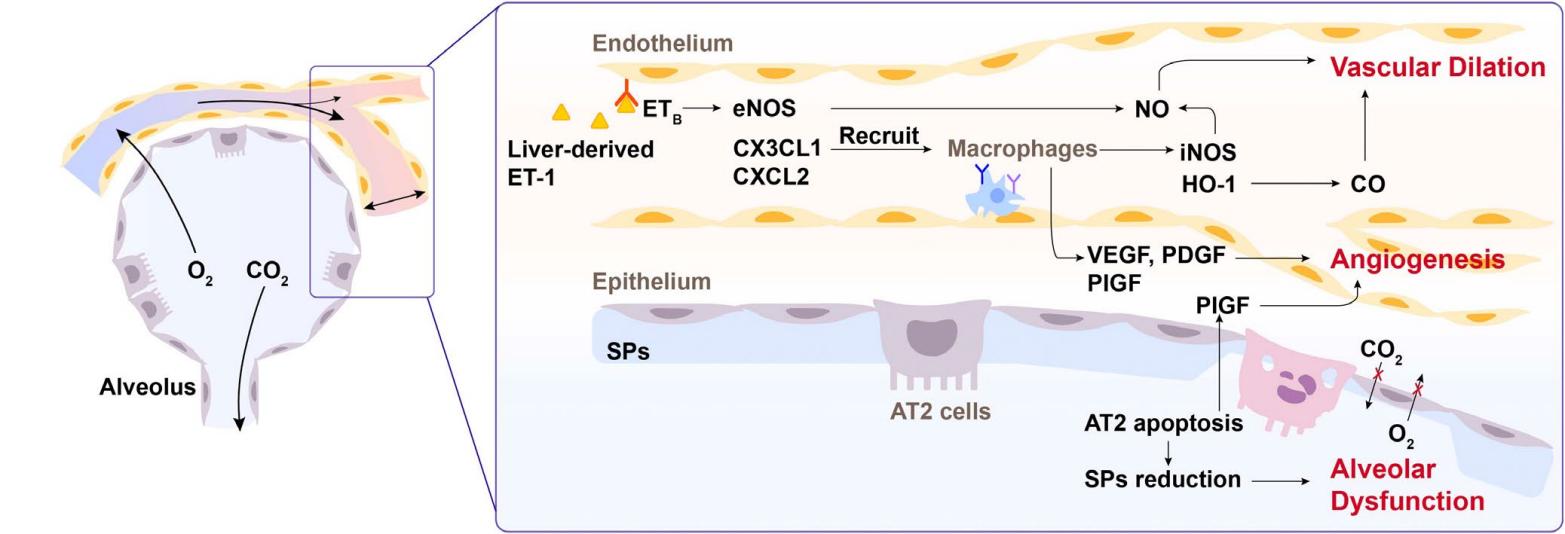
Pulmonary microenvironment disequilibrium of hepatopulmonary syndrome. ET‐1 binds to ET_B_ receptors on pulmonary endothelial cells, thereby activating eNOS and enhancing NO synthesis. Circulating monocytes are recruited by the local upregulation of chemokines, such as CX3CL1 and CXCL2, which promote the production of NO, CO, and angiogenic factors. In response to injury, AT2 cells undergo apoptosis, leading to a reduction in SPs. The resultant reduction in SPs contributes to alveolar dysfunction. AT2 cells also produce PlGF, further facilitating angiogenesis. AT2 cells, type II alveolar epithelial cells; CX3CL1, C‐X3‐C motif chemokine ligand 1; CXCL2, C‐X‐C motif chemokine ligand 2; CO, carbon monoxide; ET‐1, endothelin‐1; ET_B_ receptors, endothelin B receptors; eNOS/iNOS, endothelial/inducible NO synthase; HO‐1, heme oxygenase‐1; NO, nitric oxide; PDGF, platelet‐derived growth factor; PlGF, placental growth factor; SPs, surfactant‐associated proteins; VEGF, vascular endothelial growth factor. Created with Adobe Illustrator software.

In response to primary liver diseases, pulmonary microenvironment disequilibrium in HPS results from the abnormal adaptation and complex interactions among respiratory epithelial cells, pulmonary endothelial cells and immune cells, leading to impaired gas exchange. Clinically, three mechanisms underlie the pathogenesis of abnormal gas exchange and hypoxemia: pulmonary ventilation–perfusion mismatch, diffusion limitation and direct arteriovenous shunts [10, 16]. CBDL‐induced biliary cirrhosis in rats is the most widely studied preclinical model, as it closely mimics the features of human HPS [8, 17]. Studies on experimental models have revealed the pathophysiologic alterations underlying these three mechanisms: vascular dilation, angiogenesis and alveolar dysfunction (Figure 1).

### 2.1 Vascular Dilation

Vascular dilation refers to the abnormal widening of blood vessels. This alteration, known as IPVDs, occurs in the lungs due to liver diseases and causes blood to bypass the normal oxygen exchange process, resulting in hypoxemia. The development of IPVDs is a key feature of HPS. Endothelin‐1 (ET‐1) and nitric oxide (NO) are both key regulators of the vascular system (Figure 1). Increased hepatic and plasma ET‐1 levels in response to portal circulation changes or bile duct obstruction have been well documented in patients and rodents with liver cirrhosis, and ET‐1 correlates with gas exchange abnormalities [17, 18, 19, 20, 21]. ET‐1 is classically recognised as a potent vasoconstrictor that mediates vasoconstriction by binding to endothelin A (ET_A_) or B (ET_B_) receptors on vascular smooth muscle cells. In contrast, selective upregulation of ET_B_ receptors in pulmonary endothelial cells mediates vasodilatory responses by activating downstream endothelial NO synthase (eNOS) and enhancing NO [22, 23, 24]. Consistently, elevated exhaled NO levels have been found in cirrhotic and HPS patients, which can be normalised after liver transplantation [25, 26, 27]. Mechanistically, NO exerts a vasodilatory effect by activating the guanylate cyclase/cyclic guanosine monophosphate signalling pathway.

In addition to the ET‐1/eNOS/NO signalling pathways, intravascular accumulation of monocytes/macrophages in the lung also contributes to NO‐mediated and carbon monoxide (CO)‐mediated vascular relaxation [28, 29]. Monocytes/macrophages in the lung, potentially derived from the spleen, can induce transient upregulation of inducible NO synthase (iNOS) and activate the heme oxygenase‐1 (HO‐1)/CO system [28, 29]. Tumour necrosis factor‐alpha (TNF‐α) is a potent activator of macrophage iNOS [30]. Neutralising TNF‐α significantly improves HPS in cirrhotic rats, suggesting that targeting macrophage accumulation may be a potential strategy for HPS [31]. In HPS, infiltrating monocytes/macrophages are recruited by local upregulation of chemotactic factors and their corresponding receptors in the lung, such as C‐X3‐C motif chemokine ligand/receptor 1 (CX3CL1/CX3CR1) and C‐X‐C motif chemokine ligand/receptor 2 (CXCL2/CXCR2) [32, 33, 34]. Notably, activation of the ET‐1/ET_B_ receptor axis facilitates endothelial CX3CL1 production, linking the ET‐1/ET_B_ receptor axis to the CX3CL1/CX3CR1 axis [35]. In summary, ET‐1/eNOS/NO, iNOS/NO and HO‐1/CO signalling pathways play a critical role in the development of IPVDs and HPS. However, the primary source of ET‐1/eNOS/NO in liver cirrhosis‐induced HPS remains unclear. Whether the accumulation of monocytes/macrophages in the lung is linked to liver dysfunction remains unknown.

### 2.2 Angiogenesis

Angiogenesis refers to the formation of new vessels and capillary networks, accompanied by the activation of vascular endothelial growth factor (VEGF)‐mediated signalling pathways. Early preclinical studies observed increased capillary density in the lungs of rats with biliary cirrhosis [36]. Subsequent studies confirmed the occurrence of angiogenesis in both human and experimental HPS [37, 38, 39]. Endothelial cell proliferation and tube formation are key processes in angiogenesis. These processes can be suppressed by Annexin A1 and promoted by the bone morphogenic protein family 9 (BMP9) or microRNA [40, 41]. Pro‐angiogenic molecules and growth factors, including von Willebrand Factor (vWF), VEGF, platelet‐derived growth factor (PDGF) and placental growth factors (PlGF), are essential for angiogenesis. These pro‐angiogenic molecules have been identified in infiltrating monocytes in HPS [32, 37, 42]. Angiogenesis in experimental HPS likely results from a proinflammatory microenvironment, in which the pulmonary endothelium undergoes abnormal adaptation to inflammatory and vasoactive mediators, leading to intrapulmonary shunting and deoxygenation. Whether the lung or liver is the primary organ contributing to the proinflammatory microenvironment requires further investigation.

### 2.3 Alveolar Dysfunction

The lung interacts with the environment via a continuous epithelium in the airway. At the alveolar level, type I and type II alveolar epithelial cells (AT1/2) are crucial for maintaining lung homeostasis. In addition to pulmonary vasodilation and angiogenesis, restrictive ventilatory defects are observed in both experimental and human HPS [43, 44]. In the early stage of CBDL, increased bilirubin, endotoxins and inflammatory mediators induce pulmonary injury [45]. Alveolar epithelial cells, especially AT2 cells, undergo increased apoptosis after CBDL, reducing pulmonary surfactant‐associated proteins (SPs) and impairing alveolar integrity [43]. In contrast, AQP5 protein levels, a specific marker of AT1 cells, do not change significantly, supporting the potentially unique effect on AT2 cells [43]. Systemic inhibition of caspase‐3‐mediated pulmonary apoptosis mitigates lung injury, angiogenesis and HPS progression [45]. As caspase‐3 inactivation also protects against liver cell death and fibrogenesis, the protective role of caspase‐3 inhibitor on the HPS may partially stem from improvements in liver injury and fibrogenesis [46]. Additionally, PlGF has been shown to induce AT2 cell death during pulmonary emphysema [47]. However, aside from lung macrophages, AT2 cells are another cellular source of PlGF production during HPS, further aggravating angiogenesis [42]. This may create a vicious loop of inflammation, epithelial injury and endothelial proliferation. Restoring alveolar function may be a potential therapeutic target in HPS. The underlying mechanisms driving alveolar dysfunction should be further investigated and targeted to improve HPS effectively.

Taken together, pulmonary microenvironment disequilibrium in HPS results from the complex interaction of various lung cells. The alterations of vascular dilation, angiogenesis and alveolar dysfunction may stem from overlapping causes. Consequently, hypoxemia results from one or more of the mechanisms outlined above.

## 3 Disruption of Hepatic Sinusoidal Homeostasis

**FIGURE 2:**
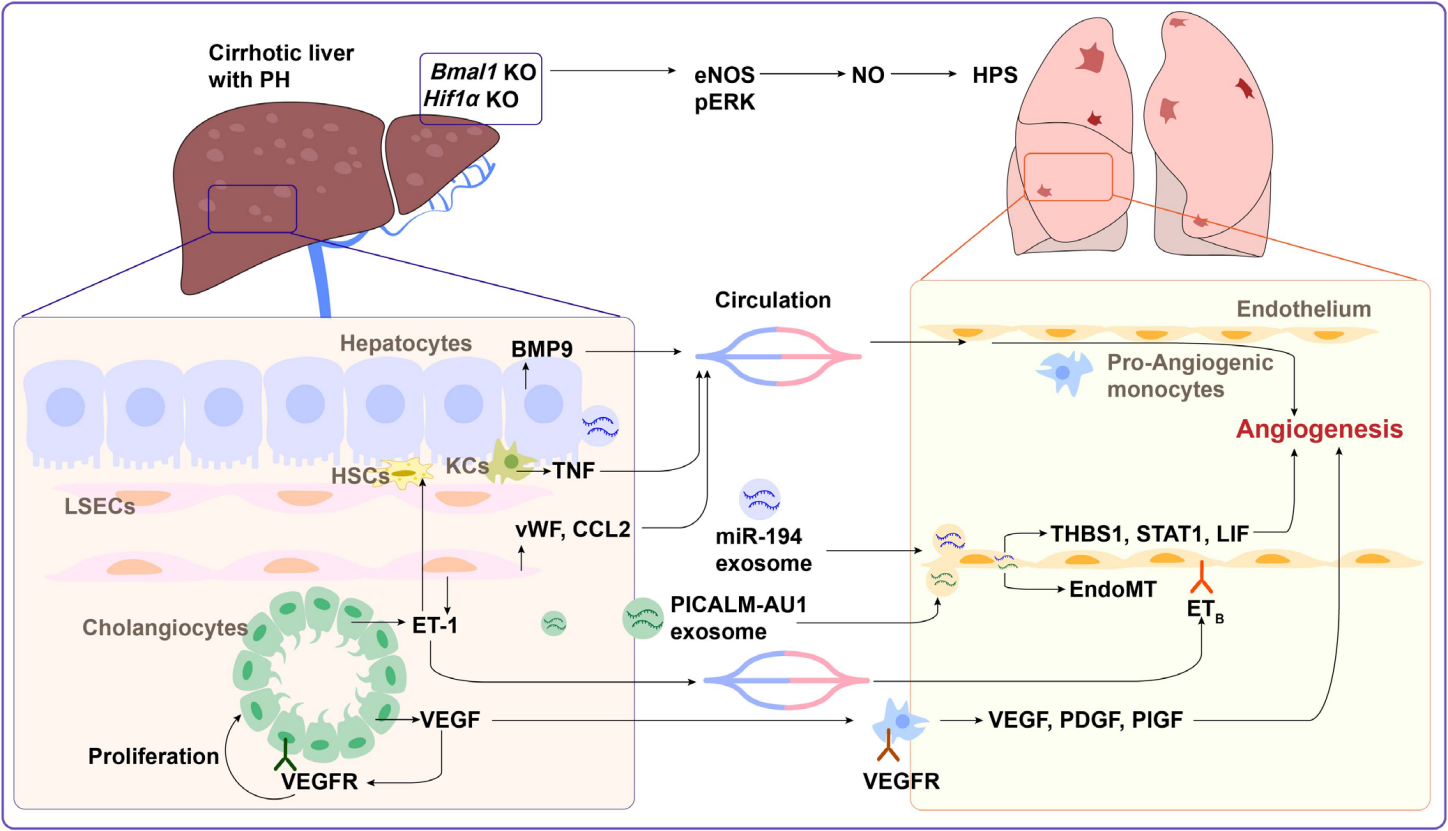
Disruption of hepatic sinusoidal homeostasis in hepatopulmonary syndrome. In liver cirrhosis, abnormal interactions between liver cells disrupt hepatic sinusoidal homeostasis. Maladaptive cells in cirrhosis ultimately induce HPS, primarily by promoting angiogenesis and monocyte infiltration in the lung. Circulating molecules and exosomes play a crucial role in mediating liver–lung communication. BMP9, bone morphogenic protein family 9; CCL2, C‐C motif chemokine ligand 2; EndoMT, endothelial–mesenchymal transition; HSCs, hepatic stellate cells; KO, knockout; KCs, Kupffer cells; LSECs, liver sinusoidal endothelial cells; PH, portal hypertension; pERK, phosphorylated extracellular signalling regulated kinase; TNF, tumour necrosis factor; vWF, von Willebrand Factor. Created with Adobe Illustrator software.

Liver cirrhosis is the primary contributor to HPS. Therefore, liver‐targeted treatments may serve as a fundamental strategy for managing HPS. Cirrhosis is a common end‐stage consequence of various etiologies. Due to the limited literature on the role of different cirrhosis‐related etiologies in developing HPS, we do not address this issue. The liver contains diverse cell types, including hepatocytes, cholangiocytes, liver sinusoidal endothelial cells (LSECs), hepatic stellate cells (HSCs), Kupffer cells (KCs) and other immune cells. These cells interact to maintain hepatic sinusoidal homeostasis under healthy conditions, which can be disrupted in liver cirrhosis [48, 49]. A recent study showed that mice lacking hepatocyte *Bmal1* and *Hif1α* develop HPS [50], highlighting the crucial liver–lung communication. Therefore, disruption of hepatic sinusoidal homeostasis may influence the progression of HPS. Herein, we provide an overview of the disruption of hepatic sinusoidal homeostasis during HPS. We begin with hepatocytes, the parenchymal cells of the liver. Next, we discuss cholangiocytes, the canonical source of ET‐1, followed by LSECs and macrophages. Given the limited literature on HSCs in this context, we do not address them in a separate section (Figure 2).

### 3.1 Hepatocytes

Hepatocytes account for approximately 60% of liver cells and play a crucial role in maintaining liver structure and function through the secretion of hepatokines and bile acids (BAs) and the clearance of vasodilators. Hepatocytes also contribute to HPS by producing C–C motif chemokine ligand 2 (CCL2), C–X–C motif chemokine ligand 1 (CXCL1), BMP9 and exosomes. Direct evidence shows that mice with hepatocyte‐specific dual deletion of *Bmal1* and *Hif1α* (*AlbCre* ^+^ *Bmal1* ^fl/fl^ *Hif1a* ^fl/fl^) develop HPS, accompanied by liver dysfunction, hypoxemia and intrapulmonary vasodilation [50]. Mechanistically, hepatocyte‐specific dual deletion of *Bmal1* and *Hif1α* results in changes to specific immune cells in the lung, increased serum levels of cytokine/chemokine (CCL2, CCL5 and CXCL1) and ultimately leads to the activation of extracellular signalling regulated kinase (ERK), as well as the accumulation of eNOS and NO in the lungs [50]. However, the key hepatokines that drive *Bmal1‐* and *Hif1α‐*dependent HPS remain unclear.

Indirect evidence also exists. BMP9 is a crucial factor in maintaining vascular quiescence, predominantly produced by the liver. The cellular source of BMP9 in the liver varies across species. BMP9 is produced in LSECs, KCs and HSCs in adult rat livers [51]. In contrast, hepatocytes and intrahepatic biliary epithelial cells are the primary source of circulating BMP9 in humans and mice [52]. Physiologically, the active form of circulating BMP9 maintains a specific level to promote vascular homeostasis by activating endothelial cells [52]. Pathologically, the circulating levels and activity of BMP are markedly decreased in cirrhotic patients and rodents with HPS [53, 54]. In a study on pulmonary arterial hypertension, another vascular complication of liver cirrhosis, BMP9 was found to modulate the endothelial synthesis/release of potent vasodilative factors, including ET‐1 [55]. Therefore, liver‐derived BMP9 plays a pleiotropic role in the liver–lung axis and supports the idea that hepatocytes contribute to HPS.

Dipeptidyl peptidase‐4 (DPP‐4), also known as CD26, is highly expressed throughout the body and plays a critical role in the development of chronic liver diseases, including those caused by hepatitis C virus infection, metabolic dysfunction‐associated steatohepatitis and hepatocellular carcinoma [56]. These conditions can be potentially managed through DPP‐4 inhibition, which exerts therapeutic effects through multiple mechanisms [56]. Vildagliptin, a DPP‐4 inhibitor, has been shown to reduce pathological vasodilation and angiogenesis in the lungs of rats with HPS, with the underlying mechanism involving the improvement of hepatic and pulmonary levels of ET‐1, eNOS, iNOS and VEGF‐A [57]. Thus, hepatoprotective‐based therapy holds promise for the treatment of HPS. Exosomes are another medium for transporting hepatocyte‐derived cargo to the lungs. Exosomal miR‐194 released from hepatocytes is internalised by pulmonary microvascular endothelial cells and promotes angiogenesis by directly targeting anti‐angiogenic factors THBS1, STAT1 and LIF [41]. Collectively, hepatocytes secrete CCL2, CXCL1, BMP9 and exosomes, which promote HPS progression. Notably, pro‐angiogenic factors, including VEGF, can also be released by injured hepatocytes and may enter circulation to exert pro‐angiogenic effects in the lung [48]. Further studies are needed to clarify whether other hepatokines are also involved in liver–lung communication during HPS.

### 3.2 Cholangiocyte

Cholangiocytes are specialised epithelial cells that line the bile ducts within the liver. Cholangiocytes contribute to HPS by producing ET‐1, VEGF and exosomes.

ET‐1 is a key vasoconstrictor that induces vascular dilation in HPS. The cellular source of ET‐1 in the liver varies depending on stimulation. In normal hepatic sinusoids, ET‐1 is primarily expressed by LSECs [58]. During the development of HPS following CBDL, cholangiocytes become the major source of ET‐1, with transforming growth factor beta 1 (TGF‐β1) serving as a potent stimulator of cholangiocyte‐derived ET‐1 [21]. Functionally, the effect of ET‐1 on blood vessels depends on the interaction between receptors and cell types. ET_A_ and ET_B_ receptors expressed on vascular smooth muscle cells induce vasoconstriction, while ET_B_ on endothelial cells induce vascular dilation by upregulating eNOS and NO [17, 23]. In HPS, ET‐1 primarily induces endothelial ET_B_ receptor/eNOS‐dependent vascular dilation [17]. Additionally, ET‐1 stimulates the production of TNF‐α and the adherence of monocytes to the lungs, thereby inducing macrophage‐dependent vascular dilation and angiogenesis, forming a vicious loop [17, 35]. Moreover, ET‐1 also exerts paracrine and autocrine effects on HSC activation [20, 58]. In preclinical settings, inhibition of the ET‐1/ET_B_ signalling pathway by targeting ET_B_ receptors significantly ameliorates HPS [23, 35]. However, further investigation is needed due to ET‐1's complex role in targeting different cell types in the liver and lungs.

Cholangiocytes also secrete VEGF and express VEGF receptors following CBDL. This autocrine mechanism promotes cholangiocyte proliferation by activating the 1,4,5‐triphosphate/Ca^2+^/protein kinase C alpha and phosphorylating Src/ERK1/2 signalling pathways [59]. In this context, excessive cholangiocyte proliferation leads to further secretion of ET‐1, followed by eNOS activation in pulmonary microvascular endothelial cells and the accumulation of intravascular monocytes [23, 35, 60]. Once cholangiocyte‐derived VEGF enters the bloodstream and is transported to the lungs, VEGF acts as a direct angiogenic factor by activating receptor tyrosine kinase in the endothelium [60]. Moreover, infiltrating macrophages can exacerbate angiogenesis by generating VEGF, PDGF, and PlGF [32, 42]. In this regard, a vicious loop forms, with cholangiocyte maladaptation as a central contributor. To counteract overactivated angiogenesis, the tyrosine kinase inhibitor sorafenib has been shown to ameliorate experimental HPS through the combination of anti‐angiogenesis effects on pulmonary endothelium and anti‐proliferation effects on cholangiocytes [11, 60]. Although a recent study confirmed an unclear benefit of sorafenib in patients with HPS [12], it remains a promising strategy to simultaneously restore hepatic and pulmonary homeostasis in HPS treatment.

Like hepatocytes, exosomes also serve as a bridge linking cholangiocytes to the lung. A long noncoding RNA PICALM‐AU1 is overexpressed by cholangiocytes and secreted as exosomes, promoting the development of HPS by mediating the endothelial–mesenchymal transition of endothelium [61]. In summary, the abnormal adaptation of cholangiocytes exhibits a crucial role in the pathophysiological process of HPS, suggesting an effective therapeutic target for HPS. However, the pivotal role of cholangiocytes needs to be confirmed in human studies and other HPS models, as the CBDL model primarily leads to cholestasis and cholangiocyte injury, which may not fully replicate human HPS.

### 3.3 LSECs

LSECs, lining the hepatic sinusoids, represent a specialised endothelial cell population characterised by a lack of organised basement membranes and the presence of open fenestrae. As previously reviewed, LSECs orchestrate sinusoidal homeostasis and contribute to liver disease progression through angiocrine signalling [48]. Angiocrine signalling refers to local cellular crosstalk between LSECs and neighbouring cells through LSEC‐derived paracrine factors [48]. These paracrine factors may enter the bloodstream due to the exclusive location of LSECs within the liver. Thus, LSECs may be the central cells connecting sinusoidal homeostasis with the lung through circulation.

LSECs act as the gatekeeper cells against liver injury and play a pivotal role in the secretion of vasoactive molecules and pro‐angiogenic factors. Dysfunction of LSECs and LSEC‐mediated angiocrine signalling may disturb the crosstalk between the liver and the lung. LSECs and HSCs are critical sources of VEGF, BMP9 and ET‐1 [48, 58, 62]. Therefore, LSECs and HSCs are thought to potentially influence HPS by regulating the concentration of VEGF, BMP9 and ET‐1 in circulation. vWF, a marker of endothelial dysfunction, is significantly elevated in patients with liver cirrhosis and portal hypertension [63]. Furthermore, patients with HPS exhibit higher levels of vWF antigen than simple‐cirrhotic patients, suggesting a close liver–lung connection and indicating that endothelial‐derived vWF could be effective for early detection of HPS [64]. Macrophage accumulation and angiogenesis are common features of liver cirrhosis and HPS. In liver fibrosis, abnormal LSEC‐mediated angiocrine signalling may lead to macrophage infiltration in the liver via the secretion of CCL2 into the plasma, which may also link hepatic LSEC and lung macrophages. LSEC‐derived angiocrine signalling in the liver may lead to increased pro‐angiogenic factors in plasma, which could drive lung angiogenesis. Although less studied, therapeutic agents targeting LSECs may offer new insights into the treatment of cirrhosis and concomitant HPS.

### 3.4 Macrophages

Hepatic macrophages are heterogeneous and play a significant role in the pathogenesis of chronic liver diseases [65, 66, 67]. In endotoxin‐induced systemic inflammation in rats, the liver and lung produce more TNF than the spleen, with KCs in the liver being the primary sources of circulating TNF [68]. During cholestasis and liver diseases, macrophages in the liver produce pro‐inflammatory mediators, including TNF [69, 70], which may explain one of the mechanisms underlying HPS development. Monocytes are recruited to the hepatic sinusoid and pulmonary intravasculature following CBDL, accompanied by a systemic elevation in monocyte numbers [29]. Bone marrow is generally considered the primary source of recruited monocytes [71]. However, the spleen is regarded as a major source of lung monocytes during HPS [29, 72]. Specific depletion of splenic monocytes by splenectomy reduced pulmonary monocyte numbers while increasing the intrahepatic monocyte infiltration and significantly impairing liver function [29]. Thus, the origin and behaviour of infiltrating monocytes in the lung and liver are distinct and tissue‐specific. Genetic lineage tracing technique and single‐cell RNA sequences may help identify the origin (spleen or bone marrow) and function (pro‐inflammatory or anti‐inflammatory, pro‐angiogenic or anti‐angiogenic) of macrophages in HPS.

Together, the abnormal adaptation of various liver cells, including hepatocytes, cholangiocytes, LSECs and macrophages, plays a significant role in both liver diseases and the progression of HPS. Of note, exosomes, a mediator for transporting liver‐derived cargo to the lung, may partially explain the cause of HPS in the context of liver cirrhosis. In addition to hepatocytes and cholangiocytes, nonparenchymal cells, another source of exosomes, require further investigation [73]. Recently, the combination of liver‐targeted and lung‐targeted therapeutic strategies for HPS treatment has gradually attracted the interest of researchers [57, 74, 75]. Besides, liver cirrhosis is the primary contributor to HPS. Thus, mice with liver cell‐specific genetic modification are promising models for delineating HPS and exploring related uncharted facets of liver‐lung communication.

## 4 Liver Dysfunction and Portal Hypertension

**FIGURE 3:**
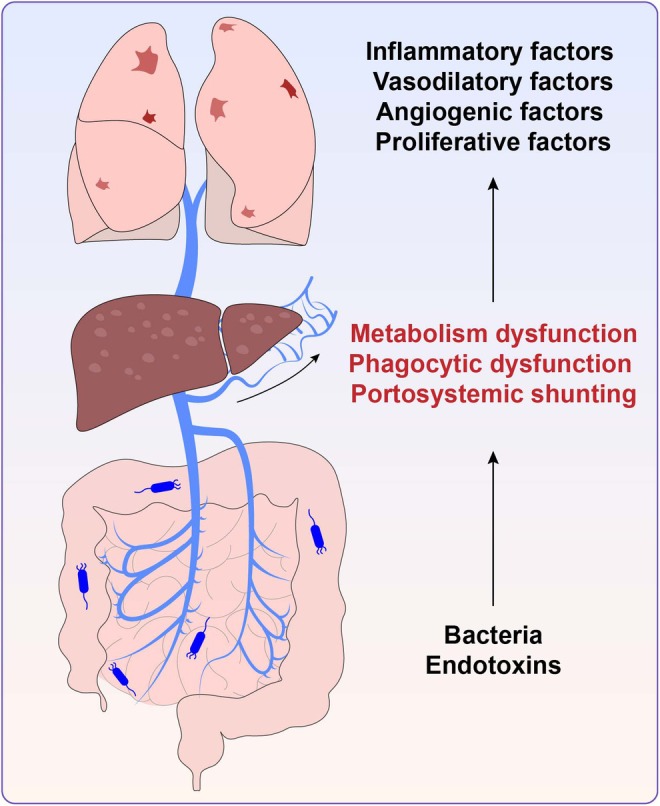
Liver dysfunction and portal hypertension promote hepatopulmonary syndrome. Liver cirrhosis and portal hypertension facilitate intestinal bacterial overgrowth and barrier disruption, resulting in bacterial translocation and endotoxemia. The development of portosystemic shunts and the decreased phagocytic capacity of the liver allow circulating bacteria or endotoxin to enter the pulmonary circulation, leading to increased levels of pulmonary inflammatory, vasodilatory, angiogenic and proliferative factors. Metabolic dysfunction of the liver has a similar effect on the lungs. The disruption of the gut–liver–lung axis, centering around the liver, facilitates the development of HPS. Created with Adobe Illustrator software.

The liver is a crucial organ for regulating metabolism and eliminating toxins and bacteria from the intestine. Notably, the relationship between the presence and severity of HPS and the severity of liver disease remains controversial. One study reported that the degree of hypoxemia in HPS is not associated with the Child‐Pugh score of liver disease [15]. However, HPS is more common in advanced cirrhosis, and HPS in patients with hypoxic hepatitis is reversible once hepatic dysfunction is normalised [76]. Thus, liver disease appears to be the primary cause of HPS development. Liver‐targeted treatment may be useful for HPS by restoring hepatic function and improving the hyperdynamic circulatory state. Thus, we hypothesise that normalising liver dysfunction and portal hypertension is crucial for resolving HPS (Figure 3).

### 4.1 Liver Dysfunction

In CBDL‐induced cirrhotic rats, liver dysfunction leads to the abnormal accumulation of metabolites, such as elevated serum oestrogen levels [77]. Accumulated oestrogen in cirrhotic rats further activates NO‐mediated intrapulmonary vascular dilation and hypoxemia [77]. The liver plays a central role in BAs metabolism [78]. Individuals with advanced cirrhosis exhibit higher concentrations of total and conjugated BAs, associated with an increased risk of organ dysfunction [79]. Notably, significant HPS occurs in CBDL‐treated rats but not in those with partial portal vein ligation (PPVL) or thioacetamide treatment [17]. In vitro studies have confirmed that the administration of major components or specific nuclear receptor agonists of BAs induces cell death and reduces the secretion of SPs in AT2 cells [43, 80]. These findings underscore the critical role of BAs in HPS. As metabolic abnormalities are a hallmark of liver cirrhosis, their role in HPS warrants further investigation.

The gut serves as a significant reservoir for bacteria and endotoxins. Bacterial translocation and endotoxemia are common in liver cirrhosis due to intestinal bacterial overgrowth, mucosal barrier disruption and decreased hepatic phagocytic capacity [81, 82]. When bacteria and endotoxins reach the pulmonary circulation, they induce the local release of chemotactic factors in the lung, recruit immune cells and activate macrophage‐mediated vascular relaxation and angiogenesis [32, 83]. In the presence of preexisting liver dysfunction induced by cholestasis, serum TNF‐α was eight times higher than that in rats with normal liver function after receiving *Escherichia coli* , despite showing equivalent circulating endotoxin levels [84]. Therefore, preexisting hepatic dysfunction may lead to excessive TNF‐α elevation induced by gut microbiota, further exacerbating lung injury. Similarly, cirrhotic rodents with HPS exhibit bacterial translocation and upregulated circulating TNF‐α and endotoxin concentration, along with the accumulation of pulmonary intravascular macrophages and the worsening of HPS and liver function [85, 86]. TNF‐α is a potent activator of macrophage iNOS/NO [30]. The recruited macrophages express iNOS, VEGF and PDGF, while macrophage depletion reverses the vasodilatory, angiogenic and proliferative effects [32]. Prevention of bacterial translocation using norfloxacine or pentoxifylline mitigates monocyte infiltration, thereby attenuating HPS [30, 83]. Inhibition of the systemic and pulmonary endotoxin/TNF‐α/NO pathway using a monoclonal antibody specific to TNF‐α significantly improves HPS and liver injury [31]. Interestingly, BAs also have a complex interaction with gut microbiota [87]. Therefore, normalising liver dysfunction, including metabolic and immune capacity, is of great significance in attenuating HPS.

### 4.2 Portal Hypertension

Portal hypertension and hyperdynamic circulation seem dispensable since PPVL rats do not develop HPS compared to CBDL rats [8], primarily because the PPVL model is non‐cirrhotic and prehepatic portal hypertension. The significant roles of circulatory changes in portal hypertension, including portosystemic shunting and shear stress, cannot be overlooked. In cirrhotic patients, portal hypertension may contribute to the development of gut dysbiosis [88]. The formation of portosystemic shunting may impair hepatic clearance of circulating bacteria or endotoxin, thereby promoting lung injury. Regarding shear stress, a previous study reported that systemic vasodilation in biliary cirrhosis depends mainly on eNOS upregulation in response to changes in shear stress [89]. Similarly, the mitigation of HPS by pentoxifylline is induced by the inhibition of TNF‐α and partially by improving the hyperdynamic circulatory state [30]. Moreover, portal hypertension significantly regulates susceptibility to ET‐1‐mediated vascular alterations [17]. In the setting of portal hypertension, the expression of the ET_B_ receptor can be regulated by changes in flow or cytokine production [90, 91, 92]. A sphingosine‐1‐phosphate (S1P) receptor agonist, fingolimod, has similar effects on reducing portal pressure, liver fibrosis, and HPS [74]. Clinically, transjugular intrahepatic portosystemic shunt creation can transiently improve deoxygenation in HPS [93, 94]. Thus, portal hypertension might play a synergistic role with the maladaptation of liver cells and liver dysfunction in the development of HPS.

Of note, chronic liver dysfunction and advanced portal hypertension are commonly induced by various etiologies, including viral infections, alcohol abuse and metabolic syndromes. Although HPS mostly occurs in cirrhotic patients, these risk factors may influence the lung and play a role in HPS, either directly or indirectly, before cirrhosis develops. A previous study established a cirrhotic rat model induced by multiple pathogenic factors and demonstrated that intestinal endotoxemia plays a central role in this HPS rat model [85]. However, adequate evaluation of the independent risks of cirrhosis‐related etiologies in HPS is still lacking both in experimental models and clinical settings. Further investigation into the potential contribution of different cirrhosis‐related etiologies to HPS is required, especially metabolic dysfunction, which is becoming the most common cause of chronic liver disease globally.

## 5 Prospective and Conclusions

HPS is a pulmonary vascular disorder complicated by liver disease and/or portal hypertension. However, no effective pharmacological therapies are currently available, and several issues remain to be solved.

First, liver‐targeted treatments may be effective for HPS. The liver is the primary contributor to HPS, and liver transplantation is the only treatment that generally reverses HPS. However, most studies and compounds have focused on targeting the lungs, yielding limited clinical benefit. Thus, exploring liver‐targeted treatments (probably a combination of previous lung‐targeted) may be a fundamental strategy for HPS. Second, new animal models are necessary. The CBDL biliary cirrhosis model has provided a pathogenic framework to further investigate pathophysiologic mechanisms and has significantly advanced our understanding of the disease. However, this model may not accurately recapitulate human liver cirrhosis‐induced HPS, as the bile ducts in human cirrhosis are not completely obstructed. New animal models that accurately recapitulate human HPS are urgently needed. Third, liver‐specific genetically modified mice and genetic lineage tracing mice, combined with multi‐omics analysis, may also help explore the mechanisms of HPS. This perspective has lacked in‐depth exploration in the past, but has recently piqued the interest of researchers. Applying emerging technologies, such as genetic modifications and multi‐omics analysis, may uncover more common hub genes underlying the liver‐lung axis and provide novel potential targets. Fourth, it is essential to evaluate the role of different cirrhosis‐related etiologies in inducing HPS, in particular metabolic factors, which are increasingly becoming the predominant cause of chronic liver disease. Finally, multicenter clinical trials in HPS are needed. Most human research on HPS involves a small number of included patients, especially case reports; therefore, providing evidence with weak strength, low quality and contradictory results. Hence, well‐designed multicenter clinical trials in HPS are needed.

In conclusion, we provide a comprehensive overview of the mechanisms and potential targets of HPS by reviewing the disruption of hepatic sinusoidal homeostasis. We present a new perspective for HPS treatment, including targeting maladaptive liver cells, liver dysfunction and portal hypertension, possibly combined with restoring pulmonary microenvironment disequilibrium.

## Author Contributions

**Jiaxin Chen:** visualization (equal), writing – original draft (equal), writing – review and editing (equal). **Yangkun Guo:** visualization (equal), writing – original draft (equal), writing – review and editing (equal). **Xiaoxun Zhang:** visualization (equal), writing – review and editing (equal). **Dengcheng Zhou:** visualization (equal), writing – review and editing (equal). **Yongfang Zhou:** visualization (equal), writing – review and editing (equal). **Qiong Pan:** visualization (equal), writing – review and editing (equal). **Jin Chai:** conceptualization (equal), project administration (equal), supervision (equal), visualization (equal), writing – original draft (equal), writing – review and editing (equal). **Jinhang Gao:** conceptualization (equal), project administration (equal), supervision (equal), visualization (equal), writing – original draft (equal), writing – review and editing (equal).

## Conflicts of Interest

The authors declare no conflicts of interest.

## Data Availability

No new data were generated during this study. All analyzed datasets are publicly available and cited appropriately.
